# Q fever in Egypt: Epidemiological survey of *Coxiella burnetii* specific antibodies in cattle, buffaloes, sheep, goats and camels

**DOI:** 10.1371/journal.pone.0192188

**Published:** 2018-02-21

**Authors:** Jessica Klemmer, John Njeru, Aya Emam, Ahmed El-Sayed, Amira A. Moawad, Klaus Henning, Mohamed A. Elbeskawy, Carola Sauter-Louis, Reinhard K. Straubinger, Heinrich Neubauer, Mohamed M. El-Diasty

**Affiliations:** 1 Institute of Bacterial Infections and Zoonoses, Friedrich-Loeffler-Institut, Jena, Germany; 2 Centre for Microbiology Research, Kenya Medical Research Institute, Nairobi, Kenya; 3 Mansoura Provincial Laboratory, Institute of Animal Health Research, Mansoura, Egypt; 4 Alshalateen Provincial Laboratory, Institute of Animal Health Research, Alshalateen, Egypt; 5 Department of Internal Medicine, Infectious Diseases and Fish Diseases, Faculty of Veterinary Medicine, Mansoura University, Mansoura, Egypt; 6 Institute of Epidemiology, Friedrich-Loeffler-Institut, Greifswald, Germany; 7 Institute of Infectious Diseases and Zoonoses, Department of Veterinary Sciences, Faculty of Veterinary Medicine, Ludwig-Maximilian University, Munich, Germany; CEA, FRANCE

## Abstract

Q fever is a zoonotic disease caused by the bacterium *Coxiella burnetii*. Clinical presentation in humans varies from asymptomatic to flu-like illness and severe sequelae may be seen. Ruminants are often sub-clinically infected or show reproductive disorders such as abortions. In Egypt, only limited data on the epidemiology of Q fever in animals are available. Using a stratified two stage random sampling approach, we evaluated the prevalence of *Coxiella burnetii* specific antibodies among ruminants and camels in 299 herds. A total of 2,699 blood samples was investigated using enzyme-linked-immunosorbent assay (ELISA). *Coxiella burnetii* specific antibodies were detected in 40.7% of camels (215/528), 19.3% of cattle (162/840), 11.2% of buffaloes (34/304), 8.9% of sheep (64/716) and 6.8% of goats (21/311), respectively. Odds of seropositivity were significantly higher for cattle (aOR: 3.17; 95% CI: 1.96–5.13) and camels (aOR: 9.75; 95% CI: 6.02–15.78). Significant differences in seropositivity were also found between domains (*Western Desert*, *Eastern Desert* and *Nile Valley and Delta*) and 25 governorates (*p* < 0.001), respectively. Animal rearing in the *Eastern Desert* domain was found to be a significant risk factor (aOR: 2.16; 95% CI: 1.62-2.88). Most seropositive animals were older than four years. No correlation between positive titers and husbandry practices or animal origin were found (*p* > 0.05). Only 8.7% of the interviewed people living on the farms consumed raw camel milk and none reported prior knowledge on Q fever. Findings from this nationwide study show that exposure to *Coxiella burnetii* is common in ruminants and camels. Disease awareness among physicians, veterinarians and animal owners has to be raised. Future epidemiological investigations have to elucidate the impact of Q fever on human health and on the economy of Egypt.

## Introduction

Q fever is a zoonotic disease in humans and animals affecting a wide range of hosts. The causative agent, *Coxiella (C*.*) burnetii*, is a Gram-negative obligate intracellular bacterium and is known for its high tenacity and infectivity [1, 2]. *C*. *burnetii* has a worldwide distribution with the exception of New Zealand [3, 4]. Q fever in humans is most often a self-limiting, flu-like illness with symptoms such as headache, myalgia or atypical pneumonia. Hepatitis or endocarditis may be long lasting sequelae in chronic cases [5–10]. Animals are often sub-clinically infected but naïve small ruminants (infected in the last trimester of gestation) may present reproductive disorders such as (late) abortion, premature delivery, stillbirth and weak offspring. Cattle often suffer from sub-clinical mastitis resulting in reduction of milk production and final break down of the quarter [11]. Ruminants shed bacteria in high numbers in birth products and to a lower extent with milk, vaginal mucus and feces or urine [12, 13]. Abortions or lambing in small ruminants have been linked to subsequent human Q fever outbreaks because birth products are heavily contaminated and can easily contaminate the environment [14, 15]. Infection in humans usually occurs via inhalation of contaminated aerosols such as dust or tick feces. In general, risk of infection is increased for people living in rural regions or with occupational risk such as people employed in veterinarian clinics, abattoirs and wool industry due to close proximity to ruminants [16, 17]. Infection risk is also elevated in areas with a high population of ruminants or movement of reservoir animals. Egypt’s hot and dry climate with little total precipitation as well as open landscapes with high wind speed may favor spreading of *C*. *burnetti* via contaminated aerosols [18]. The role of camels in transmission of *C*. *burnetii* to humans remains poorly understood [12, 19].

In Egypt like in many other developing countries, Q fever is not a notifiable disease although seroprevalences of up to 32% in adults, 22% in children and 16% in veterinarians and farmers have been reported [20–22]. Hence, a high socioeconomic impact of this disease is very likely [23].

Nevertheless, to date only limited data on the epidemiology of *C*. *burnetii* in animals are available for a few Egyptian districts although first serological evidence in Egyptian animals and humans was reported in the 1950’s [4, 17, 24–26]. Therefore, this study was carried out to describe the seroepidemiological situation of *C*. *burnetii* specific antibodies in ruminants and camels and its potential impact in Egypt (except the Sinai). This study will provide a baseline for further research into the public health impact of Q fever and implementation of public health interventions.

## Materials and methods

### Study area

The territory of the Republic of Egypt encloses over 1,001,449 km^2^ and is divided into 27 governorates. Based on its physical surface characteristics Egypt was divided into three large domains, the *Western Desert*, the *Eastern Desert*, and the *Nile Valley and Delta* region. The majority of the *Western Desert* and *Eastern Desert* domain are dry desert and steppe with scattered oasis. The *Nile Valley and Delta* region is green land with wet or muddy soil conditions. As a result of these differences in surface characteristics there is a distinct non-proportional spatial distribution of animal species and numbers within the different domains.

### Study population and study design

**Fig 1:**
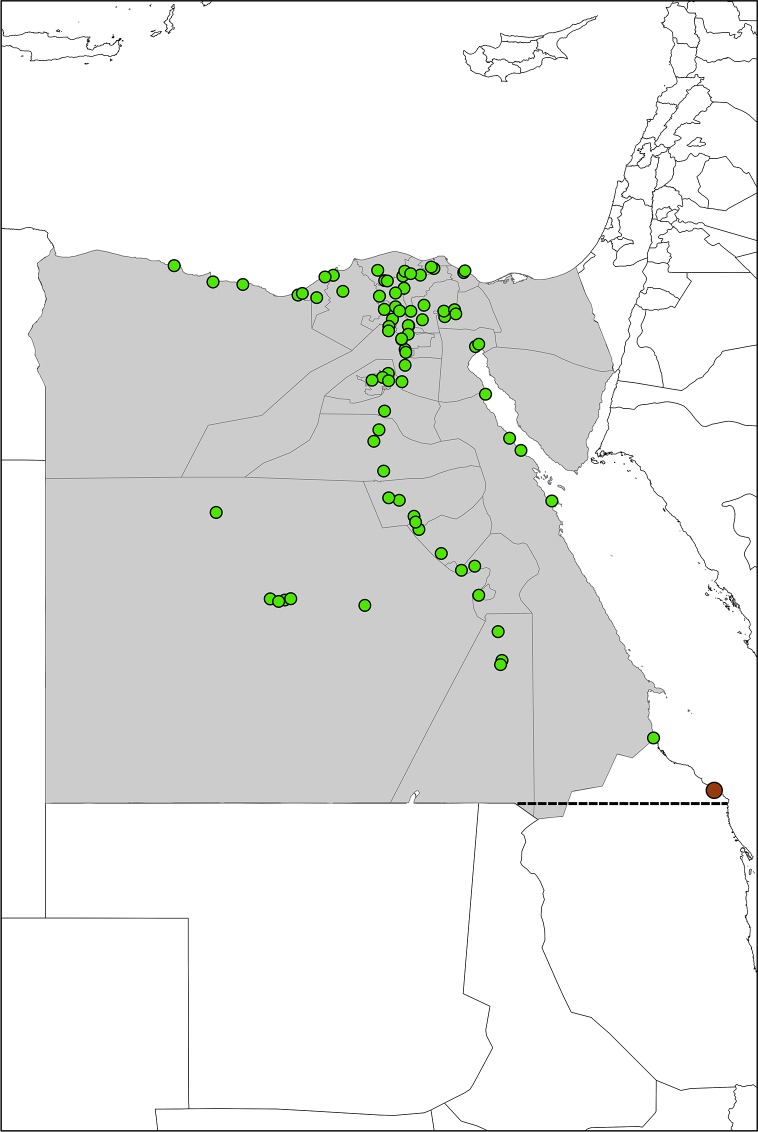
Positions of the sampled villages all over Egypt. The map of Egypt showing the position of each randomly selected sampling site (green dots) in each governorate (grey) where animals were sampled. The sampling site ‘Halayeb’, highlighted by a brown dot, is located in the territory disputed between Egypt and Sudan.

**Fig 2:**
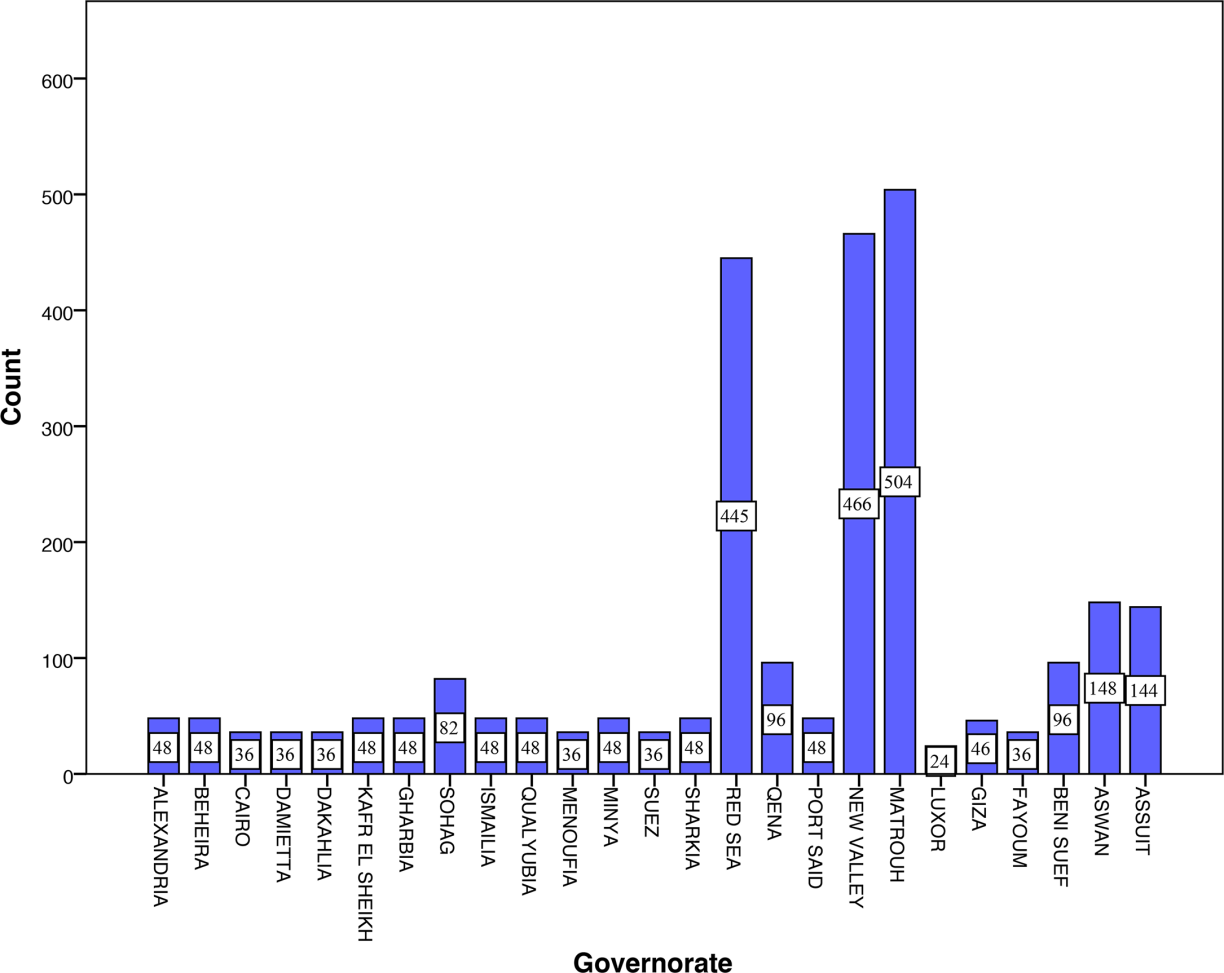
Numbers of animals sampled per governorate.

Cattle, buffalo, sheep, goat and camel herds in Egypt except those of the Sinai (governorates in the *Eastern Desert* domain) due to ongoing political and security instability were investigated. From October 2015 to March 2016 a cross-sectional study with a stratified (by governorates) two stage random cluster sampling strategy was conducted. In the first stage 80 villages were randomly selected from 25 governorates. The villages sampled are shown in Fig 1, whereas the governorates are listed in Fig 2 and S1 Table. During the second sampling stage one or two herds/farms were randomly selected without replacements from each sampling site. Thus, a total of 299 herds/farms had to be tested. Due to a full census of the village livestock population was not available sampling was distributed across all identified villages per domain. The number of animals to be tested was calculated using the two stage sampling formula. The calculated number of animals was divided by the total number of villages of each domain to obtain the final number of animals to be sampled per village. The animals sampled in the study were older than 1.5 years to avoid false positive results due to maternal antibody cross reactions in the ELISA test used. The estimated age of the animal was obtained from the farmer.

### Sample collection

Blood (5 ml) was collected from the jugular veins of sheep, goats and camels and from the tail veins (*Vena caudalis mediana*) of cattle and buffaloes. Blood samples were collected using disposable needles (18 and 19 gauges) and 50/60 ml three part syringes (AMECO, Egypt). Blood samples were then stored at room temperature for one hour to allow clotting. After centrifugation (1,449 x g, 10 minutes) serum was aliquoted into cryo-vials and stored at -20°C before being shipped to the Friedrich-Loeffler-Institut (FLI), Germany.

### Questionnaire design and data collection

A questionnaire was used to obtain information covering a wide range of factors including information about the animal (age, species, origin) and on the husbandry system practiced. The animal husbandry systems were classified as follows: (a) stable/stationary: animals were kept in an open stable with fences and a partial roof for sun protection, (b) pasture: animals were kept on pasture/steppe in a fenced area and (c) nomadic: animals ranged free, might have been guarded by a person and were occasionally moved from one area to the next. The animal owners were interviewed about their general knowledge on Q fever including transmission, clinical signs in animals and application of countermeasures such as removal of birth products to reduce risk of infection with *C*. *burnetii*. Furthermore, they were asked if they consume raw milk. The teams interviewed the respondents in Arabic language. Moreover, GPS data were determined to identify the positions of the sampled villages.

### Serological testing

The collected serum samples were screened for *C*. *burnetii* specific antibodies at the Q fever reference laboratory of the FLI. An indirect ELISA (IDEXX CHEKIT Q fever Antibody ELISA Test Kit, IDEXX Laboratories, Switzerland) was used and the results were evaluated according to the manufacturer’s recommendations. Briefly, results with an optical density (OD) of ≥40% or <30% of ($\mathrm{PK}\bar{x}‑\mathrm{NK}\bar{x}$) (PK = positive control, NK = negative control, $\bar{x}$ = mean) were considered as positive or negative, respectively. Samples with a value between ≥30% and <40% were considered equivocal and were re-tested. The manufacturer reported sensitivity and specificity of the kit to be approximately 100% [27]. The test is certified for use in sheep, goats and cattle (ruminants). Cattle and buffaloes share a closely related immune system allowing the use of this ELISA for samples from buffaloes [28]. The IDEXX ELISA is commonly used in serum samples of camels although a final validation of this test in camelids is still missing [25, 29].

### Statistical analysis

Analyses were performed using SPSS Statistics software® (Armonk, IBM Corp, USA, version 19). Missing values were coded and included in the analysis as ‘missing’. The chi-square or Fisher’s exact test was used to determine differences in seropositivity among groups categorized by age, species of the animal, location of collection, animal husbandry system and origin of the animal. Stepwise logistic regression analyses were done to examine the association between variables with p < 0.2 in univariable analysis (animal age group, animal species, origin, housing and domain) and seropositivity with adjustment for the other variables. Logistic regression models were also run for each animal species separately. Age was categorized into two groups (up to four years and over four years) and husbandry conditions were categorized into two groups (nomadic vs. ‘others’ which combined pasture, stables and missing). Odds ratios (ORs) and corresponding confidence intervals (CIs) for each category compared with the reference group were calculated. P values < 0.05 were considered significant. The map displaying the sampled villages was created using ArcGIS (ESRI, version 10).

### Ethical considerations

This study was carried out in strict accordance with the recommendations of the Egyptian Network of Research Ethics Committees (ENREC), which complies with the international laws and regulations regarding ethical considerations in research. The ENREC approved this research work. For purposes of this study all animal owners consented to sampling.

## Results

### Study population

A total of 2,699 livestock (31.1% cattle, 26.5% sheep, 19.6% camels 11.5% goats and 11.3% buffaloes) were sampled on 299 farms of 80 villages. The majority of the animals sampled was from the *Nile Valley and Delta* (47.6%) and *Western Desert* (35.9%) regions. Animals from the *Eastern Desert* domain accounted for 16.5% due to missing samples of the Sinai. Goats were only sampled in 19 of 25 governorates. The number of goat samples collected differed from the sample size calculated prior to the study, especially in the *Western Desert* and *Eastern Desert* region. One thousand six hundred and thirty-nine (60.7%) animals were nomadic, 262 (9.7%) on pasture and 685 (25.4%) stationary/stables. In the *Western Desert* region, most animals were nomadic (936/2,699 [34.7%]) whereas stationary placement (18.1%) and pasture husbandry (8.0%) were mainly found in the *Nile Valley and Delta* domain. More than eighty-eight percent (88.5%) of all sampled livestock were bred in Egypt and only 311 animals (11.5%) were imported. Camels were the only imported animals and all of them originated from Sudan (58.9% [311/528]). Nine hundred and seventy (28.5%) animals were younger than 4 years and 1,729 (64.1%) were older than 4 years. Fig 2 and Table 1 summarize the characteristics of the study population. None of the livestock owners interviewed reported prior knowledge on Q fever or on any application of countermeasures. Twenty-six owners (8.7%) reported consumption of raw camel milk. Transmission of *C*. *burnetii* to humans via consumption of raw milk is still unknown.

**Table 1:** Numbers of animals sampled per domain with age group, numbers of animals of a particular animal husbandry system and origin of animals.

Variable	Domain *n* (%)		
	Western Desert	Nile Valley a. Delta	Eastern Desert
**Animal species**			
Cattle	340 (40.5)	360 (42.9)	140 (16.7)
Buffalo	120 (39.5)	124 (40.8)	60 (19.7)
Sheep	262 (36.6)	314 (43.9)	140 (19.6)
Goat	48 (15.4)	238 (76.5)	25 (8.0)
Camel	200 (37.9)	248 (47.0)	80 (15.2)
Total	970 (35.9)	1284 (47.6)	445 (16.5)
**Animal husbandry**			
Nomadic	936 (57.1)	467 (28.5)	236 (14.4)
Pasture	34 (13.0)	215 (82.1)	13 (5.0)
Stationary/stable	0 (0)	489 (71.4)	196 (28.6)
Missing	0 (0)	113 (8.8)	0 (0)
**Origin of animal**			
Egypt	970 (40.6)	1053 (44.1)	365 (15.3)
Sudan	0 (0)	231 (74.3)	80 (25.7)
**Animal age group**			
≤ 4 years	326 (33.6)	484 (49.9)	160 (16.5)
> 4 years	644 (37.2)	800 (46.3)	285 (16.5)

n = number of animals

### Seroprevalence

The seroprevalence in goats was 6.8%, in sheep 8.9%, in buffaloes 11.2%, in cattle 19.3% and in camels 40.7% (Table 2). The differences in seroprevalence among the animal species were significant (*p* < 0.001) (Table 3). Multivariable analysis showed significantly higher odds for seropositivity for cattle (aOR: 3.17; 95% CI: 1.96–5.13) and camels (aOR: 9.75; 95% CI: 6.02-15.78) (Table 4). Cattle, sheep and camels of the *Eastern Desert* region had highest seroprevalences. Seroprevalences in buffaloes and goats were highest in the *Nile Valley and Delta* domain (p < 0.001) (Table 3). Seropositivity in the final logistic regression model was significantly associated with animals from the *Eastern Desert* domain (aOR: 2.16; 95% CI: 1.62–2.88) (Table 4). This was also evident in the analyses per animal species (S3 Table).

**Table 2:** Context of seropositivity and investigated factors of the study populations.

Variable	Total *n*	Seropositive *n* (%)	95% CI	*p* value
**Animal species**				< 0.001
Cattle	840	162 (19.3)	16.8–22.1	
Buffaloes	304	34 (11.2)	8.1–15.2	
Sheep	716	64 (8.9)	7.1–11.3	
Goats	311	21 (6.8)	4.5–10.1	
Camels	528	215 (40.7)	36.6–45.0	
**Domain**				< 0.001
Western Desert	970	165 (17.0)	14.8–19.5	
Nile Valley a. Delta	1284	211 (16.4)	14.5–18.6	
Eastern Desert	445	120 (27.0)	23.1–31.3	
**Animal husbandry**				0.002
Nomadic	1639	318 (19.4)	17.6–21.4	
Pasture	262	26 (9.9)	6.9–14.1	
Stationary/stable	685	135 (19.7)	16.9–22.9	
Missing	113	17 (15.0)	9.6–22.8	
**Origin of camels**				0.432
Egypt	217	84 (38.7)	32.5–45.3	
Sudan	311	131 (42.1)	36.8–47.7	
**Animal age group**				< 0.001
≤ 4 years	970	107 (11.0)	9.2–13.2	
> 4 years	1729	389 (22.5)	20.6–24.5	

*n* = number of animals

**Table 3:** Prevalence of *Coxiella burnetii* specific antibodies in Egyptian livestock in relation to their geographical origin.

Domain	Animal species SP [%], (95% CI)				
	Cattle	Buffaloes	Sheep	Goats	Camels
**Western Desert**	17.6 (14.0–22.1)	4.2 (1.8–9.4)	7.3 (4.7–11.1)	6.3 (2.2–16.8)	39.0 (32.5–45.9)
**Nile Valley a. Delta**	14.2 (10.9–18.2)	17.7 (12.0–25.4)	8.0 (5.5–11.5)	7.1 (4.5–11.1)	38.7 (32.9–44.9)
**Eastern Desert**	36.4 (28.9–44.7)	11.7 (5.8–22.2)	14.3 (9.4–21.0)	4.0 (0.7–19.5)	51.3 (40.5–61.9)
**Total**	19.3 (16.8–22.1)	11.2 (8.1–15.2)	8.9 (7.1–11.3)	6.8 (4.5–10.1)	40.7 (36.6–45.0)

SP = seroprevalence, CI = confidence interval

**Table 4:** Multivariable logistic regression analysis of factors associated with seropositivity.

Variable	Regression Coefficient	Standard Error	Significance	aOR	95% CI
** Animal Species**			<0.0001		
Goats^a^				1.00	
Sheep	0.21	0.27	0.429	1.23	0.73–2.07
Buffaloes	0.46	0.29	0.117	1.58	0.89–2.82
Cattle	1.15	0.24	<0.0001	3.17	1.96–5.13
Camels	2.28	0.24	<0.0001	9.75	6.02–15.78
** Domain**			<0.0001		
Western Desert^a^				1.00	
Nile Valley a. Delta	0.06	0.12	0.63	1.06	0.84–1.34
Eastern Desert	0.75	0.15	<0.0001	2.16	1.62–2.88
**Constant**	-2.74	0.25	<0.0001	0.06	

^a^reference (group with lowest risk), aOR = adjusted Odds Ratio, CI = confidence interval

Seroprevalences at governorate level ranged from 4.2% to 36.4% in cattle, from 3.3% to 100% in buffaloes, from 5.3% to 25.0% in sheep, from 4.0% to 41.7% in goats and from 12.5% to 75% in camels (p < 0.001) (S1 Table). Seroprevalences determined for the villages were in the range of 4.8%-66.7% in cattle, 4.0%-100.0% in buffaloes, 3.3%-50.0% in sheep, 8.3%-50.0% in goats and 16.7%-78.6% in camels (*p* < 0.001). Fifty-three percent (157/299) of all sampled herds had at least one seropositive animal.

Seroprevalence was found to be higher in animals from stationary/stable (135/685 [19.7%]) and nomadic (318/1639 [19.4%]) farming than from animals kept on pastures (26/262 [9.9%]) (*p* = 0.002) (Table 2). However, this variable was not significant in the multivariable analysis.

Higher seroprevalence 389/1729 (22.5%) was found in animals older than four years. Most sheep and goats with a positive result were younger than four years. The difference in seroprevalence between these age groups was statistically significant (*p* < 0.001) in the univariable analysis, but not in the multivariable analysis.

Of the imported camels 42.1% (131/311) were seropositive. Eighty-four (38.7%) camels from Egyptian origin were tested positive but the difference was not statistically significant (*p* = 0.432).

## Discussion

This first nationwide cross-sectional study in ruminants and camels was conducted to provide a deeper understanding of the epidemiology of Q fever in Egypt. An overall seroprevalence of 40.1% in camels, 19.3% in cattle, 11.2% in buffaloes, 8.9% in sheep and 6.8% in goats was found. Seroprevalences were influenced by the geographical location, type of animal husbandry and age of animal, however not by the origin of an animal. Potential risks associated with seropositivity are animal species and the geographical location. Thus, Q fever is endemic throughout Egypt in ruminants and camels.

Over the last 65 years, to the best of our knowledge, ten prevalence studies have been conducted in Egypt. These studies had limitations in study design (missing or inadequate), study area (locally restricted) or size of test specimens. Thus, all major farm animal species which might serve as natural reservoirs were investigated using reliable study design, probabilistic sampling approaches and a representative sample size. A bias may be caused by the lower number of samples collected than calculated prior to the study. This is due to the missing samples from the Sinai and a lower sample size from goats. Nevertheless, the results reported for cattle, buffaloes, sheep and camels are representative for the serprevalences in Egypt.

Gwida et al. (2014) examined dairy cattle and detected *C*. *burnetii* specific antibodies in 13.2% (158/1,194) of cattle from nine farms from Dakahlia, Damietta and Port Said governorates [24]. Their results are in agreement with the data of this study corresponding to 11.1% in these three governorates. Another study did not detect *C*. *burnetii* specific antibodies in slaughter cattle using IDEXX ELISA in central Egypt [25]. We found antibodies in livestock all across Egypt including central Egypt. Since the origin of the cattle was not defined, it may be assumed that the cattle came either from few Q fever free holdings or the animals had been recently infected and no specific antibodies had yet been produced. The same authors also failed to detect antibodies in buffaloes older than six months. We found a seroprevalence of 11.2% in buffaloes and at least one seropositive buffalo in 47.2% of buffalo herds (25/53). Our findings show that the situation in the field may have changed. The seroprevalence of *C*. *burnetii* specific antibodies in sheep in our study is in accordance with the data of a study in livestock for slaughter (8% [14/174]) [25]. In contrast, a study on farm animals from the Giza, Cairo and Fayoum governorates showed remarkably high seroprevalences in sheep and goats (32.7% [18/55] and 23.3% [7/30]), respectively [17]. This difference could be explained by the high small ruminant density of this rural region and the fact that infected small ruminants may shed bacteria in high numbers [15, 20]. In goats, the bias discussed before may also be a reason for this difference.

Aridification of many regions of Africa and Asia has increased the relevance of camels as farm animals. In Egypt, camels are kept at high numbers in dry areas for milk and meat production or as pack animals. This can result in greater transmission rates of *C*. *burnetii* and has been demonstrated in this study with the high seroprevalence found in camels. Several studies from Saudi Arabia, UAE, Iran and Chad showed that camels have antibodies against *C*. *burnetii* and are able to shed the bacteria via secretions and excretions [12, 19, 25]. Indeed, Schelling et al. (2003) found that pastoralist camel breeders in Chad had increased odds of exposure when compared to cattle breeders [19]. Thus, camels may even play the same important role in human disease as reservoir and source of *C*. *burnetii* as ruminants do.

Beside the overall seroprevalences for each animal species, significant differences in exposure were found for the domains (*p* < 0.001). Although the association between transmission and prevalence of *C*. *burnetii* is strongly influenced by landscape, climate, animal movement and high animal population density, no obvious explanations were found for these results [30]. It is likely that transmission of *C*. *burnetii* in the *Eastern Desert* domain may be favored by coastal winds. Conversely, the high seroprevalence in buffaloes in the *Nile Valley and Delta* region (aOR: 5.01; 95% CI: 1.83–13.71; S3 Table) may be explained by the high animal population density due to abundant feed and water supply. In Egypt, buffaloes are kept in stables and this may face a higher infection pressure and transmission rate. Nevertheless, further research analyzing the impact of the described factors for a significant assessment are needed.

A clear statement can be made about the influence of the type of husbandry system on seropositivity. Nomadic animal keeping did not pose a significant risk although the majority (318/496 [64.1%]) of seropositive animals were nomadic. This finding may be explained by the construction of the stables (open roofs and fences) which favor transmission of *C*. *burnetii* via aerosolisation.

*C*. *burnetii* could have also been spread by animal movements particularly during uncontrolled import of infected animals. In this survey, camels were the only livestock found to be imported to Egypt, but no correlation was found in the multivariable logistic regression. Hence, the seroprevalence found in camels from Aswan governorate bordering Sudan were strikingly high (67.5% [27/40]). A study from Iran has associated high Q fever seroprevalence in border areas with the import of infected camels [29]. A high (maybe illegal) import rate with no control may be responsible for the high seroprevalence in Aswan. Thus, the impact on transmission of *C*. *burnetii* through importation of infected animals in this region is substantial and requires immediate action to combat the potential widespread public health effects of *C*. *burnetii* on animal and human health. Measures to control the importation of camels from Sudan and Somalia to Egypt need to be implemented.

In conclusion, *C*. *burnetii* specific antibodies are present in Egyptians most important livestock species throughout the country. Especially buffaloes and camels should be the focus of any further research to establish their role in the transmission of *C*. *burnetii* to humans and to identify any potential risk factors for exposure. In African countries, a classification of husbandry systems is not expedient to identify a risk factor for *C*. *burnetii* transmission due to the open construction of stables. Whereas the specific geographical characteristics and climatic conditions may influence the seroprevalences in the Western, Eastern and Middle Egypt. Importation of animals with unknown health status has come to the fore and should be tackled immediately. Other consequences on the economy and animal and public health could not be evaluated. Nevertheless, awareness rising is needed in animal owners, veterinarians, physicians and authorities.

## Supporting information

S1 Table Prevalence of *Coxiella burnetii* specific antibodies positive tested animals in Egyptian governorates. *p* < 0.001, *n* = number, n.a. = not available.
(DOCX)

S2 Table Positive farm animals kept in different animal keeping systems in Egypt. *n* = number.
(DOCX)

S3 Table Multivariable logistic regression analyses of factors associated with seropositivity per animal species. ^a^reference (group with lowest risk), aOR = adjusted Odds Ratio, CI = confidence interval.
(DOCX)

## References

[1] Heinzen RA, Hackstadt T, Samuel JE. Developmental biology of *Coxiella burnettii*. Trends in microbiology. 1999;7(4):149–54. Epub 1999/04/28. . 10217829 10.1016/s0966-842x(99)01475-4

[2] McCaul TF, Williams JC. Developmental cycle of *Coxiella burnetii*: structure and morphogenesis of vegetative and sporogenic differentiations. Journal of bacteriology. 1981;147(3):1063–76. Epub 1981/09/01. ; PubMed Central PMCID: PMCPmc216147. 7275931 10.1128/jb.147.3.1063-1076.1981 PMC216147

[3] Hilbink F, Penrose M, Kovacova E, Kazar J. Q fever is absent from New Zealand. Int J Epidemiol. 1993;22(5):945–9. . 8282477 10.1093/ije/22.5.945

[4] Kaplan MM, Bertagna P. The geographical distribution of Q fever. Bull World Health Organ. 1955;13(5):829–60. ; PubMed Central PMCID: PMCPMC2538086. 13284560 PMC2538086

[5] Honarmand H. Q Fever: an old but still a poorly understood disease. Interdisciplinary perspectives on infectious diseases. 2012;2012:131932 Epub 2012/12/06. doi: 10.1155/2012/131932 ; PubMed Central PMCID: PMCPmc3506884. 23213331 10.1155/2012/131932 PMC3506884

[6] Derrick EH. "Q" fever, a new fever entity: clinical features, diagnosis and laboratory investigation. Reviews of infectious diseases. 1983;5(4):790–800. Epub 1983/07/01. . 6622891 10.1093/clinids/5.4.790

[7] Parker NR, Barralet JH, Bell AM. Q fever. Lancet (London, England). 2006;367(9511):679–88. Epub 2006/03/01. doi: 10.1016/s0140-6736(06)68266-4 . 16503466 10.1016/S0140-6736(06)68266-4

[8] van der Hoek W, Schneeberger PM, Oomen T, Wegdam-Blans MC, Dijkstra F, Notermans DW, et al Shifting priorities in the aftermath of a Q fever epidemic in 2007 to 2009 in The Netherlands: from acute to chronic infection. Euro surveillance: bulletin Europeen sur les maladies transmissibles = European communicable disease bulletin. 2012;17(3):20059 Epub 2012/02/03. . 22297101

[9] Raoult D, Marrie TJ, Mege JL. Natural history and pathophysiology of Q fever. The Lancet Infectious Diseases. 2005;5(4):219–26. http://dx.doi.org/10.1016/S1473-3099(05)70052-9. 15792739 10.1016/S1473-3099(05)70052-9

[10] Morroy G, van der Hoek W, Albers J, Coutinho RA, Bleeker-Rovers CP, Schneeberger PM. Population Screening for Chronic Q-Fever Seven Years after a Major Outbreak. PLoS One. 2015;10(7):e0131777 doi: 10.1371/journal.pone.0131777 ; PubMed Central PMCID: PMCPMC4489093. 26132155 10.1371/journal.pone.0131777 PMC4489093

[11] Barlow J, Rauch B, Welcome F, Kim SG, Dubovi E, Schukken Y. Association between *Coxiella burnetii* shedding in milk and subclinical mastitis in dairy cattle. Veterinary research. 2008;39(3):23 Epub 2008/02/07. doi: 10.1051/vetres:2007060 . 18252189 10.1051/vetres:2007060

[12] Mohammed OB, Jarelnabi AA, Aljumaah RS, Alshaikh MA, Bakhiet AO, Omer SA, et al *Coxiella burnetii*, the causative agent of Q fever in Saudi Arabia: molecular detection from camel and other domestic livestock. Asian Pacific Journal of Tropical Medicine. 2014;7(9):715–9. http://dx.doi.org/10.1016/S1995-7645(14)60122-X.

[13] Rodolakis A, Berri M, Hechard C, Caudron C, Souriau A, Bodier CC, et al Comparison of *Coxiella burnetii* shedding in milk of dairy bovine, caprine, and ovine herds. Journal of dairy science. 2007;90(12):5352–60. Epub 2007/11/21. doi: 10.3168/jds.2006-815 . 18024725 10.3168/jds.2006-815

[14] Maurin M, Raoult D. Q fever. Clinical microbiology reviews. 1999;12(4):518–53. Epub 1999/10/09. ; PubMed Central PMCID: PMCPmc88923. 10515901 10.1128/cmr.12.4.518 PMC88923

[15] Dijkstra F, van der Hoek W, Wijers N, Schimmer B, Rietveld A, Wijkmans CJ, et al The 2007–2010 Q fever epidemic in The Netherlands: characteristics of notified acute Q fever patients and the association with dairy goat farming. FEMS immunology and medical microbiology. 2012;64(1):3–12. Epub 2011/11/10. doi: 10.1111/j.1574-695X.2011.00876.x . 22066649 10.1111/j.1574-695X.2011.00876.x

[16] Lejeune JT, Rajala-Schultz PJ. Food safety: unpasteurized milk: a continued public health threat. Clinical infectious diseases: an official publication of the Infectious Diseases Society of America. 2009;48(1):93–100. Epub 2008/12/05. doi: 10.1086/595007 . 19053805 10.1086/595007

[17] Nahed HGK, A.-M. A. Seroprevalence of *Coxiella burnetii* antibodies among farm animals and human contacts in Egypt. Journal of American Science. 2012;8(3):619–21.

[18] Nusinovici S, Frossling J, Widgren S, Beaudeau F, Lindberg A. Q fever infection in dairy cattle herds: increased risk with high wind speed and low precipitation. Epidemiol Infect. 2015:1–11. doi: 10.1017/S0950268814003926 . 25783480 10.1017/S0950268814003926 PMC4594051

[19] Schelling E, Diguimbaye C, Daoud S, Nicolet J, Boerlin P, Tanner M, et al Brucellosis and Q-fever seroprevalences of nomadic pastoralists and their livestock in Chad. Prev Vet Med. 2003;61(4):279–93. . 14623412 10.1016/j.prevetmed.2003.08.004

[20] Abdel-Moein KA, Hamza DA. The burden of *Coxiella burnetii* among aborted dairy animals in Egypt and its public health implications. Acta tropica. 2017;166:92–5. Epub 2016/11/16. doi: 10.1016/j.actatropica.2016.11.011 . 27845064 10.1016/j.actatropica.2016.11.011

[21] Corwin A, Habib M, Olson J, Scott D, Ksiazek T, Watts DM. The prevalence of arboviral, rickettsial, and Hantaan-like viral antibody among schoolchildren in the Nile river delta of Egypt. Trans R Soc Trop Med Hyg. 1992;86(6):677–9. . 1363163 10.1016/0035-9203(92)90189-j

[22] Corwin A, Habib M, Watts D, Darwish M, Olson J, Botros B, et al Community-based prevalence profile of arboviral, rickettsial, and Hantaan-like viral antibody in the Nile River Delta of Egypt. The American journal of tropical medicine and hygiene. 1993;48(6):776–83. Epub 1993/06/01. . 8101432 10.4269/ajtmh.1993.48.776

[23] van Asseldonk MA, Prins J, Bergevoet RH. Economic assessment of Q fever in the Netherlands. Prev Vet Med. 2013;112(1–2):27–34. Epub 2013/07/23. doi: 10.1016/j.prevetmed.2013.06.002 . 23866818 10.1016/j.prevetmed.2013.06.002

[24] Gwida M, El-Ashker M, El-Diasty M, Engelhardt C, Khan I, Neubauer H. Q fever in cattle in some Egyptian Governorates: a preliminary study. BMC Res Notes. 2014;7:881 doi: 10.1186/1756-0500-7-881 ; PubMed Central PMCID: PMCPMC4295271. 25481509 10.1186/1756-0500-7-881 PMC4295271

[25] Horton KC, Wasfy M, Samaha H, Abdel-Rahman B, Safwat S, Abdel Fadeel M, et al Serosurvey for zoonotic viral and bacterial pathogens among slaughtered livestock in Egypt. Vector Borne Zoonotic Dis. 2014;14(9):633–9. doi: 10.1089/vbz.2013.1525 . 25198525 10.1089/vbz.2013.1525 PMC4676263

[26] Mazyad SA, Hafez AO. Q fever (*Coxiella burnetii*) among man and farm animals in North Sinai, Egypt. J Egypt Soc Parasitol. 2007;37(1):135–42. . 17580573

[27] IDEXX-Laboratories. Sensitivity and specificity ELISA assay 2015 [02 December 2015]. Available from: http://www2.idexx.com/view/xhtml/en_us/livestock-poultry/newsletter/2007/200708.jsf%3Bjsessionid=Lhcc8noo1efXWtKH-OKoTQ#fnq.

[28] Ibeagha-Awemu EM, Lee JW, Ibeagha AE, Zhao X. Bovine CD14 gene characterization and relationship between polymorphisms and surface expression on monocytes and polymorphonuclear neutrophils. BMC genetics. 2008;9:50 Epub 2008/08/12. doi: 10.1186/1471-2156-9-50 ; PubMed Central PMCID: PMCPmc2536669. 18691417 10.1186/1471-2156-9-50 PMC2536669

[29] Janati Pirouz H, Mohammadi G, Mehrzad J, Azizzadeh M, Nazem Shirazi MH. Seroepidemiology of Q fever in one-humped camel population in northeast Iran. Tropical animal health and production. 2015;47(7):1293–8. Epub 2015/06/14. doi: 10.1007/s11250-015-0862-z . 26070292 10.1007/s11250-015-0862-z

[30] Nusinovici S, Frossling J, Widgren S, Beaudeau F, Lindberg A. Q fever infection in dairy cattle herds: increased risk with high wind speed and low precipitation. Epidemiol Infect. 2015;143(15):3316–26. Epub 2015/03/19. doi: 10.1017/S0950268814003926 ; PubMed Central PMCID: PMCPMC4594051. 25783480 10.1017/S0950268814003926 PMC4594051

