# Supplementary material for: Q fever in Egypt: Epidemiological survey of Coxiella burnetii specific antibodies in cattle, buffaloes, sheep, goats and camels
Source: PLoS One. 2018 Feb 21;13(2):e0192188. doi: 10.1371/journal.pone.0192188 (PMC5821454; doi:10.1371/journal.pone.0192188)
Supplement: S1 Table — p < 0.001, n = number, n.a. = not available. (DOCX) [file pone.0192188.s001.docx]

| **Animal species** | **Domain** | **Governorate** (*n* total sites/*n* total farms) | **Serum positive** *n*/total [%] (95% CI) |
| --- | --- | --- | --- |
| **Cattle** | **Western Desert** | New Valley (6/6) | 26/170 [15.3] (10.66-21.47) |
|  |  | Matrouh (4/4) | 34/170 [20.0] (14.68-26.65) |
|  | **Nile Valley and Delta** | Alexandria (3/3) | 0/12 [0.0] (0.00-24.25) |
|  |  | Beheira (2/4) | 3/12 [25.0] (8.89-53.23) |
|  |  | Cairo (2/2) | 0/12 [0.0] (0.00-24.25) |
|  |  | Damietta (2/2) | 1/12 [8.3] (1.49-35.39) |
|  |  | Dakahlia (2/2) | 1/12 [8.3] (1.49-35.39) |
|  |  | Kafr El Sheikh (3/3) | 1/12 [8.3] (1.49-35.39) |
|  |  | Gharbia (3/3) | 3/12 [25.0] (8.89-53.23) |
|  |  | Sohag(2/2) | 8/24 [33.3] (17.97-53.29) |
|  |  | Ismailia (4/4) | 2/12 [16.7] (4.70-44.80) |
|  |  | Qalyubia (2/2) | 0/12 [0.0] (0.00-24.25) |
|  |  | Menoufia (3/3) | 2/12 [16.7] (4.70-44.80) |
|  |  | Minya (2/2) | 2/12 [16.7] (4.70-44.80) |
|  |  | Suez (2/2) | 4/12 [33.3] (13.81-60.94) |
|  |  | Sharkia (3/3) | 3/12 [25.0] (8.89-53.23) |
|  |  | Qena (3/3) | 0/24 [0.0] (0.00-13.80) |
|  |  | Port Said (2/2) | 2/12 [16.7] (4.70-44.80) |
|  |  | Luxor (0/0) | n.a. |
|  |  | Giza (3/3) | 1/12 [8.3] (1.49-35.39) |
|  |  | Fayoum (3/3) | 1/12 [8.3] (1.49-35.39) |
|  |  | Beni Suef (2/2) | 1/24 [4.2] (0.74-20.24) |
|  |  | Aswan (3/3) | 10/60 [16.7] (9.31-28.03) |
|  |  | Assuit (3/3) | 6/36 [16.7] (7.87-31.89) |
|  | **Eastern Desert** | Red Sea (5/5) | 51/140 [36.4] 28.92-44.66) |
|  | **Total** | 24 (69/71) | 162/840 [19.3] (16.76-22.09) |
| **Buffaloes** | **Western Desert** | New Valley (6/6) | 3/60 [5.0] (1.71-13.70) |
|  |  | Matrouh (3/3) | 2/60 [3.3] (0.92-11.36) |
|  | **Nile Valley and Delta** | Alexandria (3/3) | 2/4 [50.0] (15.00-85.00) |
|  |  | Beheira (2/2) | 1/4 [25.0] (4.56-69.94) |
|  |  | Cairo (1/1) | 0/4 [0.0] (0.00-48.99) |
|  |  | Damietta (1/1) | 0/4 [0.0] (0.00-48.99) |
|  |  | Dakahlia (1/1) | 0/4 [0.0] (0.00-48.99) |
|  |  | Kafr El Sheikh (2/2) | 2/4 [50.0] (15.00-85.00) |
|  |  | Gharbia (2/2) | 2/4 [50.0] (15.00-85.00) |
|  |  | Sohag(1/1) | 0/8 [0.0] (0.00-32.44) |
|  |  | Ismailia (2/2) | 2/4 [50.0] (15.00-85.00) |
|  |  | Qalyubia (1/1) | 1/4 [25.0] (4.56-69.94) |
|  |  | Menoufia (2/2) | 0/4 [0.0] (0.00-48.99) |
|  |  | Minya (2/2) | 0/4 [0.0] (0.00-48.99) |
|  |  | Suez (2/2) | 0/4 [0.0] (0.00-48.99) |
|  |  | Sharkia (2/2) | 4/4 [100.0] (51.01-100.00) |
|  |  | Qena (1/1) | 1/8 [12.5] (2.24-47.09) |
|  |  | Port Said (1/1) | 0/4 [0.0] (0.00-48.99) |
|  |  | Luxor (1/1) | 0/4 [0.0] (0.00-48.99) |
|  |  | Giza (2/2) | 0/4 [0.0] (0.00-48.99) |
|  |  | Fayoum (2/2) | 3/4 [75.0] (30.06-95.44) |
|  |  | Beni Suef (2/2) | 3/8 [37.5] (13.68-69.43) |
|  |  | Aswan (3/3) | 1/20 [5.0] (0.89-23.61) |
|  |  | Assuit (2/2) | 0/12 [0.0] (0.00-24.25) |
|  | **Eastern Desert** | Red Sea (6/6) | 7/60 [11.7] (5.77-22.18) |
|  | **Total** | 25 (53/53) | 34/304 [11.2] (8.11-15.22) |
| **Sheep** | **Western Desert** | New Valley (4/4) | 11/112 [9.8] (5.57-16.73) |
|  |  | Matrouh (4/4) | 8/150 [5.3] (2.73-10.17) |
|  | **Nile Valley and Delta** | Alexandria (3/3) | 0/12 [0.0] (0.00-24.25) |
|  |  | Beheira (2/2) | 2/12 [16.7] (4.70-44.80) |
|  |  | Cairo (2/2) | 1/12 [8.3] (1.49-35.39) |
|  |  | Damietta (2/2) | 3/12 [25.0] (8.89-53.23) |
|  |  | Dakahlia (2/2) | 1/12 [8.3] (1.49-35.39) |
|  |  | Kafr El Sheikh (3/3) | 0/12 [0.0] (0.00-24.25) |
|  |  | Gharbia (2/2) | 0/12 [0.0] (0.00-24.25) |
|  |  | Sohag(2/2) | 2/24 [8.3] (2.32-25.85) |
|  |  | Ismailia (4/4) | 2/12 [16.7] (4.70-44.80) |
|  |  | Qalyubia (2/2) | 1/12 [8.3] (1.49-35.39) |
|  |  | Menoufia (2/2) | 1/12 [8.3] (1.49-35.39) |
|  |  | Minya (2/2) | 1/12 [8.3] (1.49-35.39) |
|  |  | Suez (2/2) | 0/12 [0.0] (0.00-24.25) |
|  |  | Sharkia (2/2) | 2/12 [16.7] (4.70-44.80) |
|  |  | Qena (3/3) | 2/24 [8.3] (2.32-25.85) |
|  |  | Port Said (2/2) | 0/12 [0.0] (0.00-24.25) |
|  |  | Luxor (0/0) | n.a. |
|  |  | Giza (3/3) | 0/12 [0.0] (0.00-24.25) |
|  |  | Fayoum (2/2) | 3/12 [25.0] (8.89-53.23) |
|  |  | Beni Suef (2/2) | 0/24 [0.0] (0.00-13.80) |
|  |  | Aswan (2/2) | 0/14 [0.0] (0.00-21.53) |
|  |  | Assuit (3/3) | 4/36 [11.1] (4.41-25.32) |
|  | **Eastern Desert** | Red Sea (6/6) | 20/140 [14.3] (9.44-21.04) |
|  | **Total** | 24 (63/63) | 64/716 [8.9] (7.06-11.25) |
| **Goats** | **Western Desert** | New Valley (4/4) | 1/24 [4.2] (0.74-20.24) |
|  |  | Matrouh (3/3) | 2/24 [8.3] (2.32-25.85) |
|  | **Nile Valley and Delta** | Alexandria (3/3) | 2/12 [16.7] (4.70-44.80) |
|  |  | Beheira (2/2) | 1/12 [8.3] (1.49-35.39) |
|  |  | Cairo (0/0) | n.a. |
|  |  | Damietta (0/0) | n.a. |
|  |  | Dakahlia (0/0) | n.a. |
|  |  | Kafr El Sheikh (3/3) | 1/12 [8.3] (1.49-35.39) |
|  |  | Gharbia (2/2) | 3/12 [25.0] (8.89-53.23) |
|  |  | Sohag(1/1) | 0/10 [0.0] (0.00-27.75) |
|  |  | Ismailia (2/2) | 5/12 [41.7] (19.33-68.05) |
|  |  | Qalyubia (2/2) | 0/12 [0.0] (0.00-24.25) |
|  |  | Menoufia (0/0) | n.a. |
|  |  | Minya (2/2) | 1/12 [8.3] (1.49-35.39) |
|  |  | Suez (0/0) | n.a. |
|  |  | Sharkia (2/2) | 0/12 [0.0] (0.00-24.25) |
|  |  | Qena (2/2) | 0/24 [0.0] (0.00-13.80) |
|  |  | Port Said (2/2) | 0/12 [0.0] (0.00-24.25) |
|  |  | Luxor (2/2) | 0/12 [0.0] (0.00-24.25) |
|  |  | Giza (2/2) | 0/10 [0.0] (0.00-27.75) |
|  |  | Fayoum (0/0) | n.a. |
|  |  | Beni Suef (2/2) | 3/24 [12.5] (4.34-31.00) |
|  |  | Aswan (2/2) | 1/14 [7.1] (1.27-31.47) |
|  |  | Assuit (2/2) | 0/36 [0.0] (0.00-9.64) |
|  | **Eastern Desert** | Red Sea (4/4) | 1/25 [4.0] (0.71-19.54) |
|  | **Total** | 19 (45/45) | 21/311 [6.8] (4.46-10.10) |
| **Camels** | **Western Desert** | New Valley (6/6) | 41/100 [41.0] (31.87-50.80) |
|  |  | Matrouh (4/4) | 37/100 [37.0] (22.78-40.63) |
|  | **Nile Valley and Delta** | Alexandria (3/3) | 1/8 [12.5] (2.24-47.09) |
|  |  | Beheira (2/2) | 6/8 [75.0] (40.93-92.85) |
|  |  | Cairo (2/2) | 4/8 [50.0] (21.52-78.48) |
|  |  | Damietta (2/2) | 2/8 [25.0] (7.15-59.07) |
|  |  | Dakahlia (2/2) | 3/8 [37.5] (13.68-69.43) |
|  |  | Kafr El Sheikh (3/3) | 1/8 [12.5] (2.24-47.09) |
|  |  | Gharbia (3/3) | 3/8 [37.5] (13.68-69.43) |
|  |  | Sohag(2/2) | 7/16 [43.8] (23.10-66.82) |
|  |  | Ismailia (4/4) | 1/8 [12.5] (2.24-47.09) |
|  |  | Qalyubia (2/2) | 2/8 [25.0] (7.15-59.07) |
|  |  | Menoufia (2/2) | 0/8 [0.0] (0.00-32.44) |
|  |  | Minya (2/2) | 1/8 [12.5] (2.24-47.09) |
|  |  | Suez (2/2) | 4/8 [50.0] (21.52-78.48) |
|  |  | Sharkia (2/2) | 3/8 [37.5] (13.68-69.43) |
|  |  | Qena (3/3) | 4/16 [25.0] (10.18-49.50) |
|  |  | Port Said (2/2) | 2/8 [25.0] (7.15-59.07) |
|  |  | Luxor (2/2) | 4/8 [50.0] (21.52-78.48) |
|  |  | Giza (2/2) | 0/8 [0.0] (0.00-32.44) |
|  |  | Fayoum (2/2) | 3/8 [37.5] (13.68-69.43) |
|  |  | Beni Suef (2/2) | 9/16 [56.3] (33.18-76.90) |
|  |  | Aswan (3/3) | 27/40 [67.5] (52.02-79.92) |
|  |  | Assuit (3/3) | 9/24 [37.5] (21.16-57.29) |
|  | **Eastern Desert** | Red Sea (5/5) | 41/80 [51.3] (40.49-61.89) |
|  | **Total** | 25 (67/67) | 215/528 [40.7] (36.61-44.96) |
