# Supplementary material for: Q fever in Egypt: Epidemiological survey of Coxiella burnetii specific antibodies in cattle, buffaloes, sheep, goats and camels
Source: PLoS One. 2018 Feb 21;13(2):e0192188. doi: 10.1371/journal.pone.0192188 (PMC5821454; doi:10.1371/journal.pone.0192188)
Supplement: S2 Table — n = number. (DOCX) [file pone.0192188.s002.docx]

| **Animal Species** | **Animal Keeping System** *n* Serum positive [%] | | | |
| --- | --- | --- | --- | --- |
|  | **pasture** | **stationary/stable** | **nomadic** | **missing** |
| **Cattle** (*n* = 840) | 6 [0.7] | 102 [12.1] | 54 [6.4] | 0 [0.0] |
| **Buffaloes** (*n* = 304) | 0 [0.0] | 25 [8.2] | 5 [1.6] | 4 [1.3] |
| **Sheep** (*n* = 716) | 8 [1.1] | 5 [0.7] | 47 [6.6] | 4 [0.6] |
| **Goats** (*n* = 311) | 3 [1.0] | 0 [0.0] | 18 [5.8] | 0 [0.0] |
| **Camels** (*n* = 528) | 26 [1.0] | 135 [5.0] | 318 [11.8] | 17 [0.6] |
