# Supplementary material for: Q fever in Egypt: Epidemiological survey of Coxiella burnetii specific antibodies in cattle, buffaloes, sheep, goats and camels
Source: PLoS One. 2018 Feb 21;13(2):e0192188. doi: 10.1371/journal.pone.0192188 (PMC5821454; doi:10.1371/journal.pone.0192188)
Supplement: S3 Table — areference (group with lowest risk), aOR = adjusted Odds Ratio, CI = confidence interval. (DOCX) [file pone.0192188.s003.docx]

| **Cattle** | | | | | |
| --- | --- | --- | --- | --- | --- |
| **Variable** | **Regression Coefficient** | **Standard Error** | **Significance** | **aOR** | **95% CI** |
| **Domain** |  |  | <0.0001 |  |  |
| Western Desert^a^ |  |  |  | 1.00 |  |
| Nile Valley a. Delta | -0.27 | 0.21 | 0.200 | 0.77 | 0.51-1.15 |
| Eastern Desert | 1.01 | 0.23 | <0.0001 | 2.75 | 1.76-4.29 |
| **Constant** | -1.52 | 0.14 | <0.0001 |  |  |
|  |  |  |  |  |  |
| **Buffaloes** | | | | | |
| **Variable** | **Regression Coefficient** | **Standard Error** | **Significance** | **aOR** | **95% CI** |
| **Domain** |  |  | 0.007 |  |  |
| Western Desert^a^ |  |  |  | 1.00 |  |
| Nile Valley a. Delta | 1.61 | 0.51 | 0.002 | 5.01 | 1.83-13.71 |
| Eastern Desert | 1.11 | 0.61 | 0.068 | 3.04 | 0.92-10.01 |
| **Constant** | -3.14 | 0.46 | <0.0001 |  |  |
|  |  |  |  |  |  |
| **Sheep** | | | | | |
| **Variable** | **Regression Coefficient** | **Standard Error** | **Significance** | **aOR** | **95% CI** |
| **Domain** |  |  | 0.052 |  |  |
| Western Desert^a^ |  |  |  | 1.00 |  |
| Nile Valley a. Delta | 0.11 | 0.32 | 0.725 | 1.12 | 0.60-2.08 |
| Eastern Desert | 0.76 | 0.34 | 0.026 | 2.13 | 1.10-4.14 |
| **Constant** | -2.55 | 0.29 | <0.0001 |  |  |
|  |  |  |  |  |  |
| **Goats** | | | | | |
| **Variable** | **Regression Coefficient** | **Standard Error** | **Significance** | **aOR** | **95% CI** |
| **Constant** | -2.62 | 0.23 | 0.073 |  |  |
|  |  |  |  |  |  |
| **Camels** | | | | | |
| **Variable** | **Regression Coefficient** | **Standard Error** | **Significance** | **aOR** | **95% CI** |
| **Housing** |  |  | 0.002 |  |  |
| other^a^ |  |  |  | 1.00 |  |
| nomadic | 0.86 | 0.28 | 0.002 | 2.37 | 1.36-4.12 |
| **Age category** |  |  | 0.024 |  |  |
| ≤ 4 years^a^ |  |  |  | 1.00 |  |
| > 4 years | -0.75 | 0.33 | 0.024 | 0.47 | 0.25-0.91 |
| **Constant** | -0.38 | 0.36 | 0.290 |  |  |
